# A descriptive analysis of published case reports of severe allergic reactions induced by rituximab

**DOI:** 10.1097/MD.0000000000049513

**Published:** 2026-07-03

**Authors:** Lijialong Yuan, Lixia Teng, Ziwei Deng, Ronghui Yuan, Xiang Zhao, Zhihua Shi, Yueping Jiang

**Affiliations:** a Department of Clinical Pharmacy, Hunan University of Medicine General Hospital, Huaihua, Hunan, China; b Department of Clinical Pharmacy, People’s Hospital of Chenxi, Huaihua, Hunan, China; c Department of General Practice, Hunan University of Medicine General Hospital, Huaihua, Hunan, China; d Department of Pharmacy, Xiangya Hospital, Central South University, Changsha, Hunan, China; e National Clinical Research Center for Geriatric Disorders, Xiangya Hospital, Central South University, Changsha, Hunan, China.

**Keywords:** adverse drug reaction, allergic reaction, anaphylaxis, clinical features, rituximab

## Abstract

This study aimed to characterize the clinical profile of severe allergic reactions associated with rituximab therapy. We conducted a descriptive, retrospective analysis of published case reports and case series. Relevant reports published between January 2000 and October 2025 were identified through searches of PubMed, Embase, China National Knowledge Infrastructure, Chinese Science and Technology Journal Database, and WanFang databases. Data on patient demographics, clinical presentation, management, and outcomes were extracted and analyzed descriptively. A total of 24 documented cases were included. Patients had a mean age of 46.4 years, with a range of 6 to 86 years. Females accounted for 54.2% of the cases. The most frequent underlying condition was hematological malignancy, reported in 58.3% of patients. The majority of allergic reactions occurred within 24 hours of infusion, and nearly half occurred during the initial administration. Notably, in the included cases, 75.0% of patients developed severe reactions despite the administration of standard premedication. Predominant symptoms were dyspnea (37.5%), chest tightness (29.2%), laryngeal edema (25.0%), chills (20.8%), and hypotension (16.7%). According to the World Allergy Organization criteria, 75.0% of the cases met the diagnostic criteria for anaphylaxis. Primary management strategies included discontinuation of rituximab (79.2%), corticosteroids (54.1%), epinephrine (37.5%), and antihistamines (37.5%). While most patients (83.3%) improved following intervention, 20.8% required care in an intensive care unit or emergency department. Causality assessment using the Naranjo scale rated the association as “probable” in 58.3% of cases and “certain” in 25.0%. Rituximab can induce severe and potentially life-threatening allergic reactions. These reactions may occur in patients of any age, during any treatment cycle, and even after prophylactic medication has been administered. Most reactions are acute in onset and primarily involve the respiratory and cardiovascular systems. These findings highlight the importance of close monitoring and prompt intervention during rituximab therapy. Further studies are needed to refine preventive and management strategies.

## 1. Introduction

Rituximab is a chimeric anti-cluster of differentiation 20 (CD20) monoclonal antibody derived from both human and murine components. It is widely used to treat various B-cell-mediated diseases, including non-Hodgkin lymphoma, chronic lymphocytic leukemia, rheumatoid arthritis, and several autoimmune disorders.^[1]^ Rituximab selectively binds to CD20 antigens expressed on the surface of B cells and exerts therapeutic effects through antibody-dependent cellular cytotoxicity, complement-dependent cytotoxicity, and apoptosis. Despite its established efficacy, rituximab has been associated with a range of adverse drug reactions, including severe allergic or hypersensitivity reactions. These reactions may be life-threatening and can interrupt or delay treatment. Therefore, early recognition and prompt clinical intervention are essential. This study aimed to analyze the clinical characteristics of severe allergic reactions induced by rituximab and to provide evidence to support the safe administration of this medication.

## 2. Materials and methods

### 2.1. Study design

This study was a descriptive, retrospective analysis of published case reports and case series. No original patient data were collected. The study was not designed to estimate incidence or establish causal inference beyond the associations reported in the included publications.

### 2.2. Literature search strategy

We systematically searched PubMed, Embase, China National Knowledge Infrastructure, Chinese Science and Technology Journal Database, and WanFang databases for case reports and case series published between January 2000 and October 2025. No language restrictions were applied; both English and Chinese publications were included. The search strategy incorporated the following terms and their Chinese equivalents: “Rituximab,” “MabThera,” “allerg*,” “hypersensitivity,” “anaphylaxis,” “shock,” “利妥昔单抗,” “美罗华,” “过敏,” “超敏,” “休克.” In addition, the reference lists of relevant reviews and included articles were manually screened to identify further eligible cases.

### 2.3. Inclusion and exclusion criteria

Eligible cases were selected according to the inclusion and exclusion criteria described below.

#### 2.3.1. Inclusion criteria

1. **Study type:** Published case reports or case series reporting one or more patients.
2. **Drug exposure:** The patient received rituximab (including biosimilars or the brand name MabThera®) for any indication.
3. **Adverse event:** The report clearly described an allergic or hypersensitivity reaction attributed to rituximab, including at least one of the following: clinical symptoms (e.g., dyspnea, hypotension, urticaria, angioedema, laryngeal edema), anaphylaxis diagnosed according to established criteria, or a documented allergic reaction requiring medical intervention.
4. **Clinical details:** The report provided sufficient information to extract the patient’s age or sex, the characteristics of the reaction (e.g., timing, symptoms), and the outcome (e.g., improvement, no improvement, death).
5. **Language:** English or Chinese.
6. **Publication period:** January 2000 to October 2025.

### 2.3.2. Exclusion criteria

1. **Literature type:** Review articles, editorials, letters without original case data, expert commentaries, preclinical studies, basic research, or animal studies.
2. **Trial data:** Clinical trials (e.g., randomized controlled trials, phase I – intravenous studies) that report only aggregate incidence data without individual case-level details.
3. **Uncertain attribution:** Case reports in which rituximab was not clearly identified as the suspected causative agent or in which an alternative cause of the allergic reaction, such as concomitant medication, radiocontrast exposure, or blood transfusion, was identified.
4. **Insufficient description:** Reports that mentioned the allergic reaction only vaguely (e.g., “mild infusion reaction” or “tolerated well”) without specific symptom details.
5. **Duplicate publications:** The same patient appeared in multiple articles; only the most complete version was retained.
6. **Full-text unavailability:** The full text could not be obtained after contacting the corresponding author or through interlibrary loan.

### 2.4. Literature screening and data extraction

**Figure 1.**
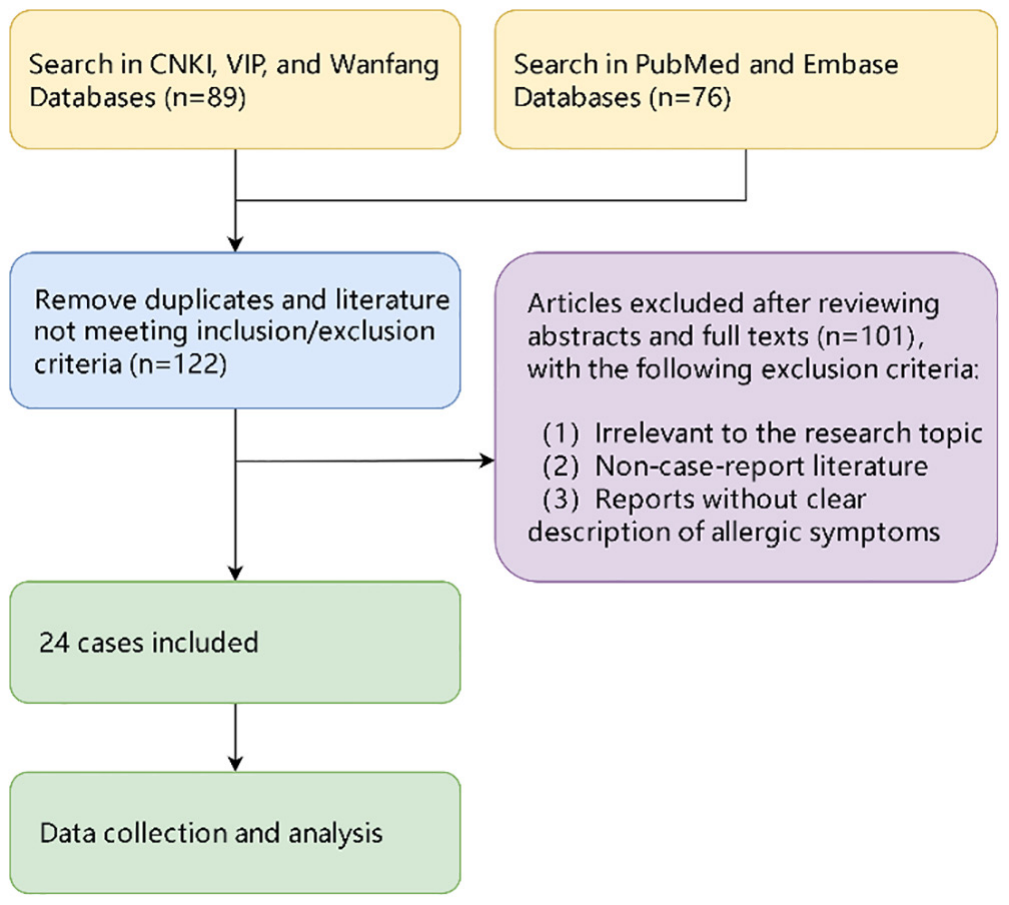
Flow diagram of literature selection.

Two investigators independently screened the literature according to the inclusion and exclusion criteria described in Section 2.3. Titles and abstracts were first reviewed, followed by full-text assessment of potentially eligible articles. Any disagreements during the screening process were resolved through consensus discussion or, when necessary, by consulting a third researcher (Fig. 1). From the included reports, we extracted data on patient demographics, including sex and age; clinical information, including indication for rituximab, dosage, and premedication status; reaction characteristics, including infusion cycle at onset, time to onset, and specific symptoms; management measures; abnormal laboratory findings; requirement for intensive care unit (ICU) or emergency department admission; final outcome; and causality assessment of the adverse reaction.

### 2.5. Data analysis

All extracted data were organized using Microsoft Excel 2024 and analyzed descriptively. To systematically classify the severity of allergic reactions in the included cases, we adopted the World Allergy Organization (WAO) 2020 anaphylaxis diagnostic criteria as the primary classification framework.^[2]^ According to the WAO 2020 criteria, anaphylaxis is diagnosed when either of the 2 criteria listed in Table 1 is met. Causality was assessed using the Naranjo Adverse Drug Reaction Probability Scale, which categorizes associations as “doubtful,” “possible,” “probable,” or “certain.” Two reviewers independently applied the scale to each case. Disagreements were resolved through discussion or consultation with a third reviewer.

**Table 1:** WAO 2020 diagnostic criteria for anaphylaxis. Adapted from Cardona et al^[2]^ World Allergy Organization (WAO) 2020 anaphylaxis diagnostic criteria.

Criterion	Description
Criterion 1	Acute onset of an illness (minutes to several hours) with involvement of the skin, mucosal tissue, or both (e.g., generalized hives, pruritus or flushing, swollen lips-tongue-uvula) AND at least one of the following: Respiratory compromise (e.g., dyspnea, wheeze-bronchospasm, stridor, hypoxemia) Reduced blood pressure or associated symptoms of end-organ dysfunction (e.g., hypotonia, syncope, incontinence) Severe gastrointestinal symptoms (e.g., severe crampy abdominal pain,repetitive vomiting), especially after exposure to non-food allergens
Criterion 2	Acute onset of hypotension or bronchospasm or laryngeal involvement after exposure to a known or highly probable allergen for that patient (minutes to several hours), even in the absence of typical skin involvement

WAO = World Allergy Organization.

## 3. Results

### 3.1. Baseline characteristics of included patients

A total of 24 cases were included in the final analysis. Among them, 11 patients were male and 13 were female, corresponding to 45.8% and 54.2%, respectively.^[3–23]^ Patient age ranged from 6 to 86 years, with a mean age of 46.4 years and a median age of 53 years. The study population included 3 pediatric patients aged 18 years or younger,^[2,4,10]^ 16 adult patients aged 19 to 64 years,^[3,7–9,11,12,14,16,17,19–22]^ and 4 elderly patients aged 65 years or older.^[5,13,19,23]^ Age was not reported in one case.^[15]^ Hematological diseases, mainly lymphoma and leukemia, were the most common indications for rituximab therapy, accounting for 12 cases (Table 2).^[5,7,8,15–17,19–22]^

**Table 2:** Baseline characteristics of the included cases.

Case No.	Sex	Age (yr)	Indication	Allergy history	Causality assessment*	Reference
1	Male	53	Pemphigus	Not reported	Probable	^[3]^
2	Male	6	Membranous nephropathy	Not reported	Certain	^[4]^
3	Female	86	Lymphoma	No known allergy	Certain	^[5]^
4	Female	8	Nephrotic syndrome	No known allergy	Probable	^[6]^
5	Female	56	Chronic lymphocytic leukemia	No known allergy	Probable*	^[7]^
6	Female	58	Rheumatoid arthritis, Sjögren syndrome	Not reported	Probable	^[8]^
7	Female	53	Marginal zone lymphoma	Not reported	Certain	^[8]^
8	Male	61	Marginal zone lymphoma	Not reported	Probable	^[8]^
9	Female	53	Basal artery epidermolysis acantholytica	Not reported	Possible	^[9]^
10	Male	12	Pemphigus	Not reported	Certain	^[10]^
11	Female	58	Rheumatoid arthritis	No known allergy	Certain	^[11]^
12	Female	59	Non-Hodgkin lymphoma	No known allergy	Possible	^[12]^
13	Male	80	Non-Hodgkin lymphoma	Not reported	Possible	^[13]^
14	Female	23	Lupus	Not reported	Probable	^[14]^
15	Female	Not reported	Non-Hodgkin B-cell lymphoma	Not reported	Certain	^[15]^
16	Male	48	Diffuse large B-cell lymphoma	No known allergy	Probable	^[16]^
17	Female	46	Follicular B-cell lymphoma	No known allergy	Probable	^[17]^
18	Female	39	Non-Hodgkin lymphoma	No known allergy	Probable	^[17]^
19	Female	66	ANCA-associated glomerulonephritis	Not reported	Probable	^[18]^
20	Female	19	Post-transplant lymphoproliferative disorder	No known allergy	Probable	^[19]^
21	Male	41	Lymphoma	Not reported	Possible	^[20]^
22	Male	28	burkitt leukemia	Not reported	Probable	^[21]^
23	Male	41	Acute lymphoblastic leukemia	Not reported	Probable	^[22]^
24	Male	65	Thyroid tumor	Not reported	Probable	^[23]^

ANCA = anti-neutrophil cytoplasmic antibody.

* Causality assessment was performed using the Naranjo scale.

### 3.2. Drug administration and timing of allergic reactions

Among the 24 cases analyzed, rituximab dosage varied. A standard regimen of 375 mg/m^2^ weekly was used in 5 cases,^[4–7,12]^ fixed intravenous doses of 1 gram or 1.5 gram were used in 4 cases,^[3,9,10,23]^ and a low dose of 100 mg or 600 mg was used in 5 cases.^[18–22]^ Dosage information was not reported in the remaining 10 cases.^[8,11,13–17]^ Most allergic reactions were acute in onset. Fifteen cases, accounting for 62.5%, occurred within 24 hours of rituximab infusion,^[5–8,10,16–21,23]^ often within 30 to 60 minutes, and presented with acute symptoms such as dyspnea and hypotension. Delayed reactions, defined as those occurring 24 hours or more after infusion, were reported in 2 cases,^[12,14]^ accounting for 8.3%. These reactions occurred 1 day and 3.5 weeks after infusion, respectively, and included presentations consistent with allergic pneumonitis. The time to onset was not documented in 7 cases,^[3,4,9,11,13,15,22]^ accounting for 29.2%. Severe allergic reactions occurred both during the first infusion and after multiple infusions. Eleven patients, or 45.8%, experienced reactions during the first infusion,^[6,8,10,11,13,16,17,19,21,22]^ whereas 12 patients, or 50.0%, developed reactions during subsequent infusions.^[3–5,7–9,14,15,18,20,23]^ These findings suggest that the risk of severe allergic reactions may persist throughout the course of rituximab therapy (Table 3).

**Table 3:** Clinical characteristics of the 24 patients included in the case reports and case series.

Parameter	Category	Value, n (%) or median (range)
Age (yr)		53 (6, 86)
Sex	Male	11 (45.8%)
	Female	13 (54.2%)
Indication	Hematological diseases (e.g., lymphoma, leukemia)	14 (58.3%)
	Dermatological diseases (e.g., pemphigus)	2 (8.3%)
	Renal diseases (e.g., nephropathy, nephritis)	3 (12.5%)
	Other/not reported	5 (20.8%)
Dosage	1 g or 1.5 g IV	4 (16.7%)
	100 mg or 600 mg IV	5 (20.8%)
	375 mg/m^2^ IV	5 (20.8%)
	Not reported	10 (41.6%)
Infusion cycle at reaction	First infusion	11 (45.8%)
	Subsequent infusions	12 (50.0%)
	Not reported	1 (4.2%)
Time to reaction onset	<24 h	15 (62.5%)
	≥24 h	2 (8.3%)
	Not reported	7 (29.2%)
Required ICU/ED admission	Yes	5 (20.8%)
	Not reported	19 (79.2%)
Outcome	Improved	20 (83.3%)
	Not improved	3 (12.5%)
	Not reported	1 (4.1%)
History of drug/food allergy	No known allergy	9 (37.5%)
	Not reported	15 (62.5%)
Causality assessment	Certain	6 (25.0%)
	Probable	14 (58.3%)
	Possible	4 (16.7%)

ED = emergency department, ICU = intensive care unit, IV = intravenous.

### 3.3. Management, outcome, and causality assessment

Five patients, accounting for 20.8%, required treatment in an ICU or emergency department.^[3,5,11,13,14]^ Most patients improved after short-term management, with clinical improvement reported in 20 of 24 cases,^[3–8,13–24]^ or 83.3%. In contrast, 3 patients, accounting for 12.5%, showed no improvement,^[9–11]^ and outcome data were unavailable in one case.^[12]^ Causality assessment using the Naranjo scale rated the association between rituximab and the allergic reaction as “probable” in 14 cases,^[3,6–8,14,16–19,21–23]^ accounting for 58.3%; “certain” in 6 cases,^[4,5,8,10,11,15]^ accounting for 25.0%; and “possible” in 4 cases,^[9,12,13,20]^ accounting for 16.7% (Tables 2 and 3).

### 3.4. Premedication status

Premedication use was explicitly reported in 18 patients,^[3,4,7–11,13,16–22]^ accounting for 75.0% of the included cases. Premedication status was not reported in the remaining 6 cases.^[5,6,12,14,15,23]^ The occurrence of severe reactions despite premedication indicates that routine prophylactic medication does not completely eliminate the risk of rituximab-induced severe allergic reactions (Table 4).

**Table 4:** Clinical presentation and management of severe allergic reactions.

Parameter	Category	Value, n (%)
Premedication	Yes	18 (75.0%)
	Not reported	6 (25.0%)
Concomitant medications	Yes	10 (41.7%)
	Not reported	14 (58.3%)
Past medical history	Relevant history reported	5 (20.8%)
	Not reported	19 (79.2%)
Prodromal symptoms	Present	6 (25.0%)
	Absent/Not reported	18 (75.0%)
Management measures	Drug discontinuation	19 (79.2%)
	Epinephrine	9 (37.5%)
	Corticosteroids	13 (54.1%)
	Antihistamines	9 (37.5%)
	Not reported	3 (12.5%)
Common clinical symptoms	Hypotension	4 (16.7%)
	Dyspnea	9 (37.5%)
	Laryngeal edema	6 (25.0%)
	Rash	4 (16.6%)
	Chills	5 (20.8%)
	Chest tightness	7 (29.2%)
Severe clinical manifestations	Confusion	1 (4.2%)
	Hypertensive crisis	1 (4.2%)
	Pulmonary edema	1 (4.2%)
Met WAO anaphylaxis criteria	Yes	18 (75.0%)
	No	6 (25.0%)
Abnormal laboratory findings	Elevated IgE	1 (4.2%)
	Abnormal complement (C3/C4)	2 (8.3%)
	Elevated tryptase	2 (8.3%)
	Abnormal heart rate	7 (29.2%)
	Abnormal blood pressure	6 (25.0%)
	Abnormal oxygen saturation	6 (25.0%)
	Elevated C-reactive protein	3 (12.5%)
	Elevated troponin	1 (4.2%)

C3 = complement component 3, C4 = complement component 4, WAO = World Allergy Organization.

### 3.5. Prodromal symptoms

Prodromal symptoms were documented in 6 patients,^[3,4,8,15,18,23]^ accounting for 25.0% of cases. Reported manifestations included cutaneous symptoms, such as rash or skin erosion, as well as headache, fever, palpitations, facial flushing, tachypnea, and tachycardia (see Table 4).

### 3.6. Clinical manifestations of allergic reactions

The included cases showed that rituximab-associated severe allergic reactions can involve multiple organ systems. Respiratory symptoms were the most prominent, with dyspnea reported in 9 patients (37.5%) and laryngeal edema in 6 patients (25.0%).^[4,7,8,10–12,16,17,19,23]^ Chest tightness was reported in 7 cases (29.2%).^[6,8,16,17,23]^ Cardiovascular involvement was indicated by hypotension in 4 patients (16.7%),^[3,5,6,10]^ while systemic symptoms such as chills occurred in 5 patients (20.8%).^[17,20,21,23]^ Cutaneous manifestations, mainly rash, were observed in 4 patients (16.6%).^[10,11,14,18]^ In addition to the primary symptoms, other clinical features include angioedema, arthralgia, fever, and numbness in the oral region or upper limbs. More severe clinical presentations were also noted in individual cases, including confusion,^[15]^ hypertensive crisis, and pulmonary edema.^[13]^ According to the WAO anaphylaxis criteria, 18 of the 24 cases, accounting for 75.0%,^[3–8,10,11,13,15–19,22,23]^ met the diagnostic criteria for anaphylaxis (Table 4).

### 3.7. Management measures

The primary management measure was discontinuation of rituximab, which was reported in 19 cases,^[3–10,16–23]^ accounting for 79.2%. Additional pharmacological interventions included corticosteroids, such as dexamethasone, in 13 cases,^[4,7,9,11,16–23]^ accounting for 54.1%; epinephrine in 9 cases,^[3,4,6–8,11,16,17,22]^ accounting for 37.5%; and antihistamines, such as famotidine, in 9 cases,^[5–8,11,17–20]^ accounting for 37.5%. Management details were not reported in 3 cases,^[12,14,15]^ accounting for 12.5% (Table 4).

## 4. Discussion

We analyzed 24 published cases of severe allergic reactions induced by rituximab. Although the median patient age was 53 years, the age range was broad, extending from 6 to 86 years and including pediatric, adult, and elderly patients. This finding suggests that rituximab-induced severe allergic reactions can occur across a wide age range and are not limited to a specific age group. Although females accounted for a slightly higher proportion of cases, the limited number of included reports precludes any conclusion regarding sex as an independent risk factor. Hematological malignancies, particularly lymphoma and leukemia, were the most common indications for rituximab therapy in the included cases. This finding is consistent with the widespread use of rituximab in hematologic diseases. Previous studies have reported that the incidence of hypersensitivity reactions among patients with non-Hodgkin lymphoma ranges from 3.9 to 8.4%,^[24]^ underscoring the need for heightened vigilance in this population. In the present analysis, most severe reactions occurred within 24 hours of infusion, with many developing during or shortly after the initial administration. These rapid-onset reactions usually present as acute infusion reactions manifesting as hypotension, fever, chills, urticaria, dyspnea, and laryngeal edema, with clinical features consistent with an immunoglobulin E (IgE)-mediated type I hypersensitivity mechanism.^[25]^ Notably, a substantial proportion of reactions (10 patients, 41.7% of those with documented timing relative to the infusion cycle) emerged after multiple doses, implying a possible link to cumulative exposure or treatment duration, as previously suggested.^[26]^ Allergic reactions to rituximab can develop after any initial or subsequent exposure, necessitating vigilance across all treatment cycles. Delayed reactions, defined as those occurring 24 hours or more after administration, were observed in 8.3% of cases. These reactions typically present as arthralgia or rash, which is a clinical picture consistent with serum sickness. Serum sickness is a type III hypersensitivity reaction that characteristically manifests between 1 and 30 days after exposure.^[27]^ In the included cases, severe allergic reactions occurred in 18 out of 24 patients despite documented premedication administration. This observation indicates that premedication does not guarantee prevention of severe reactions, although the lack of a comparator group (e.g., patients who received premedication and did not react) precludes any conclusion about its overall efficacy. Therefore, a more individualized preventive strategy may be necessary for high-risk patients. One reason for this prophylactic failure could be that mechanisms beyond mediator inhibition, such as complement activation or cytokine release, are involved and are not adequately targeted by conventional antihistamines and corticosteroids.^[28]^ Severe reactions to rituximab are often multifactorial, involving a cascade of immune mediators, such as IL‑6, IL‑8, and TNF‑α, which are not adequately addressed by conventional premedication.^[29]^

In clinical practice, rituximab-induced allergic reactions have been found to mainly involve the respiratory, cardiovascular, and cutaneous systems. The most common symptoms include dyspnea, laryngeal edema, chest tightness, hypotension, chills, and rashes. Although anaphylaxis was not explicitly mentioned in the drug insert, our study revealed that these symptoms often occur simultaneously or sequentially. Without timely discontinuation of the drug and administration of antiallergic treatment, patients’ lives were at risk of entering a critical state. The occurrence of extreme manifestations such as confusion, hypertensive crisis, and pulmonary edema in a few cases, together with the observation that 75.0% of cases met the established anaphylaxis criteria,^[2]^ reinforces the potentially fatal nature of these reactions. While most patients improved with short‑term management, the fact that 20.8% required ICU/ER admission indicates that a subset of patients experienced reactions severe enough to necessitate advanced support.

The pathogenesis of rituximab-induced allergies has not been fully elucidated. Currently, 2 main mechanisms have been proposed. Vasodilation, bronchospasm, and hypotension in rapid-onset reactions are primarily mediated by IgE. Cross-linking of IgE by rituximab on mast cells and basophils causes degranulation. This, in turn, triggers the release of histamine, leukotrienes, and other chemical mediators. However, delayed reactions typically occur through different pathways. Mechanisms such as complement activation, cytokine release syndrome, and direct cytotoxicity fall into this non-IgE mediated category. The binding of rituximab to CD20 on B cells may trigger cytokine release, leading to allergy-like symptoms.^[30]^ The absence of prodromal symptoms in most cases (75.0%), in contrast to their presence in others, further illustrates the individual variability and unpredictability of reaction onset.

Although this study was not designed to definitively identify risk factors, several associations can be inferred. Allergic reactions occurred across various dosage regimens (standard, fixed, low), and the dosage was not reported in nearly half of the cases, suggesting that dose alone is not the sole determinant. Although the infusion rate was not directly evaluated here, rapid infusion is a recognized risk factor for monoclonal antibody infusion reactions.^[30]^ Furthermore, the underlying disease status, concomitant medications, and genetic predispositions likely influence individual susceptibility and severity.^[31]^ Causality was assessed as “Probable” or “Certain” in over 83% of cases, strongly supporting the association with rituximab. Future research should prioritize large-scale clinical studies designed to identify and validate independent risk factors. Such efforts will form the basis for more accurate risk assessment and stratified patient management.

Several limitations should be acknowledged. First, this study is a descriptive analysis of published case reports, which are subject to publication bias; severe, unusual, or successfully managed cases are more likely to be reported, while mild or typical reactions may be underreported. Second, a substantial proportion of cases had missing data on key variables, including exact dosage (45.8%), infusion rate (100% missing), time to onset (29.2%), and premedication details (25.0%), limiting our ability to perform stratified analyses. Third, the absence of a denominator population precludes any estimation of incidence or relative risk. Fourth, the heterogeneity in reporting quality across case reports may have introduced information bias. Fifth, causality assessment using the Naranjo scale is inherently subjective to some degree despite independent review. Therefore, our findings should be interpreted as hypothesis-generating rather than confirmatory, and further large-scale, prospective pharmacovigilance studies are needed to validate these observations.

## 5. Conclusion

Rituximab can induce severe hypersensitivity reactions. Anaphylaxis represents a life-threatening condition. These reactions have been reported in patients of all ages and can occur during any treatment cycle. Standard premedication regimens do not consistently prevent severe reactions in all cases, as demonstrated by the occurrence of reactions in premedicated patients in this case series. The clinical presentation is typically rapid. The respiratory and cardiovascular systems are commonly and severely affected. Therefore, clinicians should be vigilant in the use of rituximab and should be aware of each patient’s specific allergy history and risk profile to prepare for anaphylaxis. Further research on the allergic mechanism of this drug is still an urgent issue, and such research will contribute to the development of more reliable prediction tools and effective prevention strategies.

## Authors contributions

**Conceptualization:** Zhihua Shi, Yueping Jiang.

**Data curation:** Lijialong Yuan, Lixia Teng, Ronghui Yuan.

**Formal analysis:** Lijialong Yuan, Lixia Teng, Ronghui Yuan.

**Methodology:** Xiang Zhao.

**Writing – original draft:** Lijialong Yuan.

**Writing – review & editing:** Ziwei Deng, Xiang Zhao.
